# A Novel Plant Leaf Patch Absorbed With IL-33 Antibody Decreases Venous Neointimal hyperplasia

**DOI:** 10.3389/fbioe.2021.742285

**Published:** 2021-10-29

**Authors:** Boao Xie, Xiche Bai, Peng Sun, Liwei Zhang, Shunbo Wei, Hualong Bai

**Affiliations:** ^1^ Department of Vascular and Endovascular Surgery, First Affiliated Hospital of Zhengzhou University, Zhengzhou, China; ^2^ Key Vascular Physiology and Applied Research Laboratory of Zhengzhou City, Zhengzhou, China; ^3^ The First Zhongyuan Middle School, Zhengzhou, China

**Keywords:** plant leaf, rapamycin, neointimal hyperplasia, IL-33, patch

## Abstract

**Introduction:** We recently showed that a decellularized leaf scaffold can be loaded with polylactic-co-glycolic acid (PLGA)-based rapamycin nanoparticles, this leaf patch can then inhibit venous neointimal hyperplasia in a rat inferior vena cava (IVC) venoplasty model. IL-33 plays a role in the neointimal formation after vascular injury. We hypothesized that plant leaves can absorb therapeutic drug solution and can be used as a patch with drug delivery capability, and plant leaves absorbed with IL-33 antibody can decrease venous neointimal hyperplasia in the rat IVC venoplasty model.

**Method:** A human spiral saphenous vein (SVG) graft implanted in the popliteal vein was harvested from a patient with trauma and analyzed by immunofluorescence. Male Sprague-Dawley rats (aged 6–8 weeks) were used to create the IVC patch venoplasty model. Plant leaves absorbed with rhodamine, distilled water (control), rapamycin, IL-33, and IL-33 antibody were cut into patches (3 × 1.5 mm^2^) and implanted into the rat IVC. Patches were explanted at day 14 for analysis.

**Result:** At day 14, in the patch absorbed with rhodamine group, immunofluorescence showed rhodamine fluorescence in the neointima, inside the patch, and in the adventitia. There was a significantly thinner neointima in the plant patch absorbed with rapamycin (*p* = 0.0231) compared to the patch absorbed with distilled water. There was a significantly large number of IL-33 (*p* = 0.006) and IL-1β (*p* = 0.012) positive cells in the human SVG neointima compared to the human great saphenous vein. In rats, there was a significantly thinner neointima, a smaller number of IL-33 (*p* = 0.0006) and IL-1β (*p* = 0.0008) positive cells in the IL-33 antibody-absorbed patch group compared to the IL-33-absorbed patch group.

**Conclusion:** We found that the natural absorption capability of plant leaves means they can absorb drug solution efficiently and can also be used as a novel drug delivery system and venous patch. IL-33 plays a role in venous neointimal hyperplasia both in humans and rats; neutralization of IL-33 by IL-33 antibody can be a therapeutic method to decrease venous neointimal hyperplasia.

## Introduction

Plants and animals are from two different kingdoms, it is rare and always unbelievable that part of a plant can be used as a substitute in animals. But several recent studies showed some exciting results on plant-based scaffolds *in vitro* experiments; decellularized spinach and parsley can be decellularized with human endothelial cells, the authors showed that decellularized plants can be scaffolds in tissue engineering (Gershlak et al., 2017). Decellularized spinach leaf scaffolds can also accelerate stem cell growth and differentiation in bone tissue engineering (Salehi et al., 2020). Recently, a group decellularized three different plant tissues (apple, carrot, and celery), examined their properties (porosity, mechanical properties), and explored their potential application in the regeneration of different tissues *in vitro* (Contessi Negrini et al., 2020).

We recently showed that a decellularized leaf scaffold can be loaded with polylactic-co-glycolic acid (PLGA)-based rapamycin nanoparticles, these nanoparticle-perfused leaves could inhibit venous neointimal hyperplasia in a rat inferior vena cava (IVC) venoplasty model at day 14; decellularized onion cellulose can also be coated with PLGA rapamycin nanoparticles and inhibit venous neointimal hyperplasia (Bai et al., 2021a). Plant leaf has a very specific architecture, including leaf stem, leaf midrib, vascular system, vein, and cuticle (Fan et al., 2019; Zhao et al., 2020). This system is important for water and nutrient transportation and storage. Recent development of biomaterials, like heparin-bonded covered stents, human acellular vessels, and tissue-engineered vascular grafts from human-induced pluripotent stem cells, greatly contributed to the advancement of vascular surgery (Lammer et al., 2013; Niklason and Lawson, 2020; Luo et al., 2020); drug-coated stents and balloons also contributed to a better clinical result; but there are limitations in surface coating and the fact that drugs and therapeutic agents could not be completely secured and delivered to the diseased sites, thus leading to the waste of treatment drugs or sometimes failure of the treatment (Iglesias et al., 2019; Ali et al., 2019). So new methods to deliver drugs are needed. It is commonly known that plant leaves can absorb and store water from the leaf stem, but there is also water loss from the leaf; the cumulative area of the stomatal pores is typically less than 3% of the leaf area, stomatal transpiration of leaves is a dominant pathway of plant physiological water loss; the leaf transpiration rate when stomata are fully open is commonly at the same level as the evaporation rate; and the rapid evaporation from the leaf surface can accelerate the absorption of the water from the leaf stem (Buckley et al., 2017; Xu et al., 2019;). We hypothesized that leaves can be used as a novel drug delivery system by their absorption capability.

IL-33 is a new member of the IL-1 family, but it is has not been well investigated in vascular surgery (Pinto et al., 2018). One study showed that IL-33 plays a role in neointimal formation after vascular injury (Govatati et al., 2020). In a mouse femoral artery endothelium denudation model by wire injury, IL-33 expression was required for neointima formation (Govatati et al., 2019). We also hypothesized that neutralization of IL-33 by IL-33 antibody can decrease venous neointimal hyperplasia.

Based on these previously findings, we hypothesized that plant leaves can absorb therapeutic drug solution and act as a novel drug delivery system, and plant leaves absorbed with IL-33 antibody can decrease venous neointimal hyperplasia in a rat IVC venoplasty model.

## Methods

The study was approved by the Animal Care and Use Committee of the First Affiliated Hospital of Zhengzhou University. All animal care procedures complied with the Guide for the Care and Use of Laboratory Animals. NIH guidelines for the Care and Use of Laboratory Animals (NIH Publication #85-23 Rev. 1985) were followed.

### Plant Leaf Absorption

**FIGURE 1:**
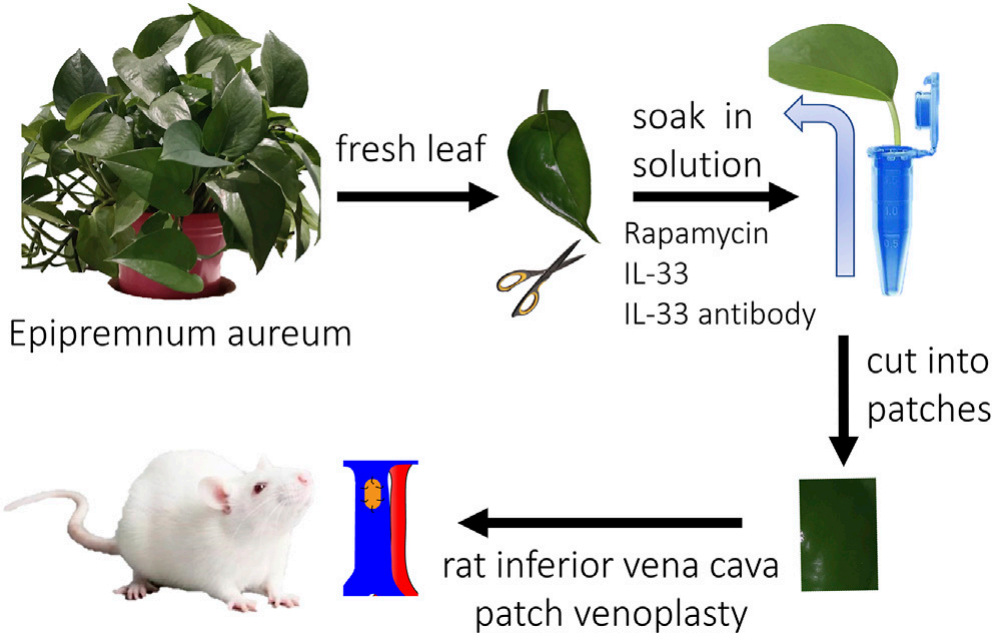
Illustration photograph showing the study design (This illustration was created by HB).

Fresh plant leaves (Epipremnum aureum, a common indoor plant in China) were harvested with an intact leaf stem and washed by running water, then the leaves were rinsed with distilled water (pH value, 6.0). The leaves were put into 1.5 ml Eppendorf tubes with distilled water, rhodamine solution (1:200, wt/wt), rapamycin solution (500 μg/500 ul, 7130031, Solarbio, China), IL-33 solution (P6317, 100 μg/500 ul, Beyotime, China), and IL-33 antibody (100 μg/500 ul, AF3626, R&D, United States) solution at room temperature for 2 days. In the rhodamine group, a color change was observed routinely; in the other groups, the plant leaves were harvested after 2 days and cut into patches for implantation **(** Figure 1 **)**.

To observe the amount of drugs in the leaf or patch, a leaf was put into 1.5 ml Eppendorf tube with distilled water for 2 days, the amount of water that leaf absorbed was calculated (389.0 ± 10.21 μl, mean ± SEM, original amount of water-remaining amount of water); the area of the leaf was measured by Image J (19.31 ± 0.5869 cm^2^, mean ± SEM), then the density of the drug (20.2 ± 0.9018 μl/cm^2^, mean ± SEM) was calculated; the size of the patch was 3 × 1.5 x 0.6 mm. Finally, the amount of the drug in the leaf patch could be calculated; rapamycin, 909.0 ± 40.58 ng/patch; IL-33 (181.8 ± 8.117 ng/patch); IL-33 antibody (181.8 ± 8.117 ng/patch).

### Human Samples

There is frequently thick neointimal hyperplasia and a high rate of restenosis after venous procedures (Bai et al., 2017a). IL-33 plays a role in the neointimal formation after vascular injury (Govatati et al., 2020), so we examined IL-33 expression in the human venous neointima. Human samples were obtained as described previously (Bai et al., 2020a). Briefly, a trauma patient required popliteal vein reconstruction with a spiral saphenous vein graft (SVG) in the popliteal vein, amputation was performed on day 18 after vascular reconstruction because of the patient’s serious injuries, and all protocols involving human biospecimens complied with all relevant ethical regulations. Tissues were processed and stained as described previously (Bai et al., 2020a), briefly, the SVG was fixed and embedded in paraffin and sectioned (4 μm thickness); sections were heated in citric acid buffer (pH 6.0) for antigen retrieval, then the sections were incubated with primary antibody overnight (Table 1). The sections were incubated with appropriate secondary antibodies (Table 1) for 1 h at room temperature and counterstained with DAPI (Solarbio, Beijing, China) (Bai et al., 2020a). The great saphenous vein (GSV) was used as control.

**TABLE 1:** Antibodies used in this experiment.

Antibodies	Vendor	Lot number	Concentration
Primary antibody			
CD34	Abcam	Ab81289	1:100
Nestin	Abcam	Ab11306	1:100
CD68	Abcam	Ab31360	1:100
α-actin	Abcam	Ab5694	1:200
CD3	Santa Cruz	Sc-20047	1:100
CD45	Santa Cruz	Sc-1178	1:100
Cleaved caspase-3	Cell signaling	9661	1:50
PCNA	Abcam	Ab29	1:100
IL-33	R&D	AF3626	1:100
IL-1β	R&D	AF-401-NA	1:100
Secondary antibody			
Goat anti-rabbit	Bioworld	BS12478	1:100
Goat anti-mouse	Bioworld	BS13278	1:100
488 goat anti-mouse	Abclone	AS073	1:100
CY3 goat anti-rabbit	Abclone	AS007	1:100

### Animal Model

Male Sprague-Dawley rats (aged 6–8 weeks) were used. The IVC patch venoplasty model was performed as previously described (Bai et al., 2017b). The rhodamine group (rhodamine solution absorption), control group (only distilled water absorption), rapamycin group (rapamycin solution absorption), IL-33 group (IL-33 solution absorption), and IL-33 antibody group (IL-33 antibody solution absorption) patches (approximately 3 × 1.5 mm^2^) were implanted into the rat infrarenal IVC using continuous 10–0 nylon sutures; there were three animals in each group. Rats were sacrificed on postoperative day 14, and the patches were explanted for analysis (Figure 1). No immunosuppressive agents, antibiotics, antiplatelet agents, or heparin were administered at any time.

### Histology Staining

Rats were anesthetized with an intraperitoneal injection of 10% chloral hydrate, and tissues were fixed with a transcardial perfusion of PBS followed by that of 10% formalin. Tissue was removed and fixed overnight in 10% formalin followed by a 24-h immersion in 70% alcohol. Tissue was then embedded in paraffin and sectioned (4 μm thickness). Tissue sections were deparaffinized and stained with H&E stain (Baso, Zhuhai, China) according to the manufacturer’s recommendations. Four random representative high power fields were counted from each patch and the mean number of cells in each high power field was recorded. Neointimal and adventitial thickness were the mean of measurements from the surface edge to the edge of the patch in three independent areas (Bai et al., 2016).

### Immunohistochemistry

Sections were heated in citric acid buffer (pH 6.0, Beyotime, Shanghai, China) at 100°C for 10 min for antigen retrieval. Sections were then treated with 0.3% hydrogen peroxide for 30 min and incubated overnight at 4°C with primary antibodies (Table 1). After overnight incubation, the sections were incubated with appropriate secondary antibodies (Table 1) for 1 h at room temperature and treated with a 3,3N-diaminobenzidine tetrahydrochloride horseradish peroxidase Color Development Kit (Beyotime, Shanghai, China) to detect the reaction products. Finally, the sections were counterstained with hematoxylin (Baso, Zhuhai, China).

### Immunofluorescence

Tissue sections were deparaffinized and then incubated with primary antibodies (Table 1) overnight at 4°C. The sections were incubated with secondary antibodies (Table 1) for 1 h at room temperature; subsequently, sections were stained with DAPI (Solarbio, Beijing, China) to stain cellular nuclei. For the rhodamine patch, the sections were deparaffinized and counterstained with DAPI, then immediately observed under a fluorescence microscope.

## Results

**FIGURE 2:**
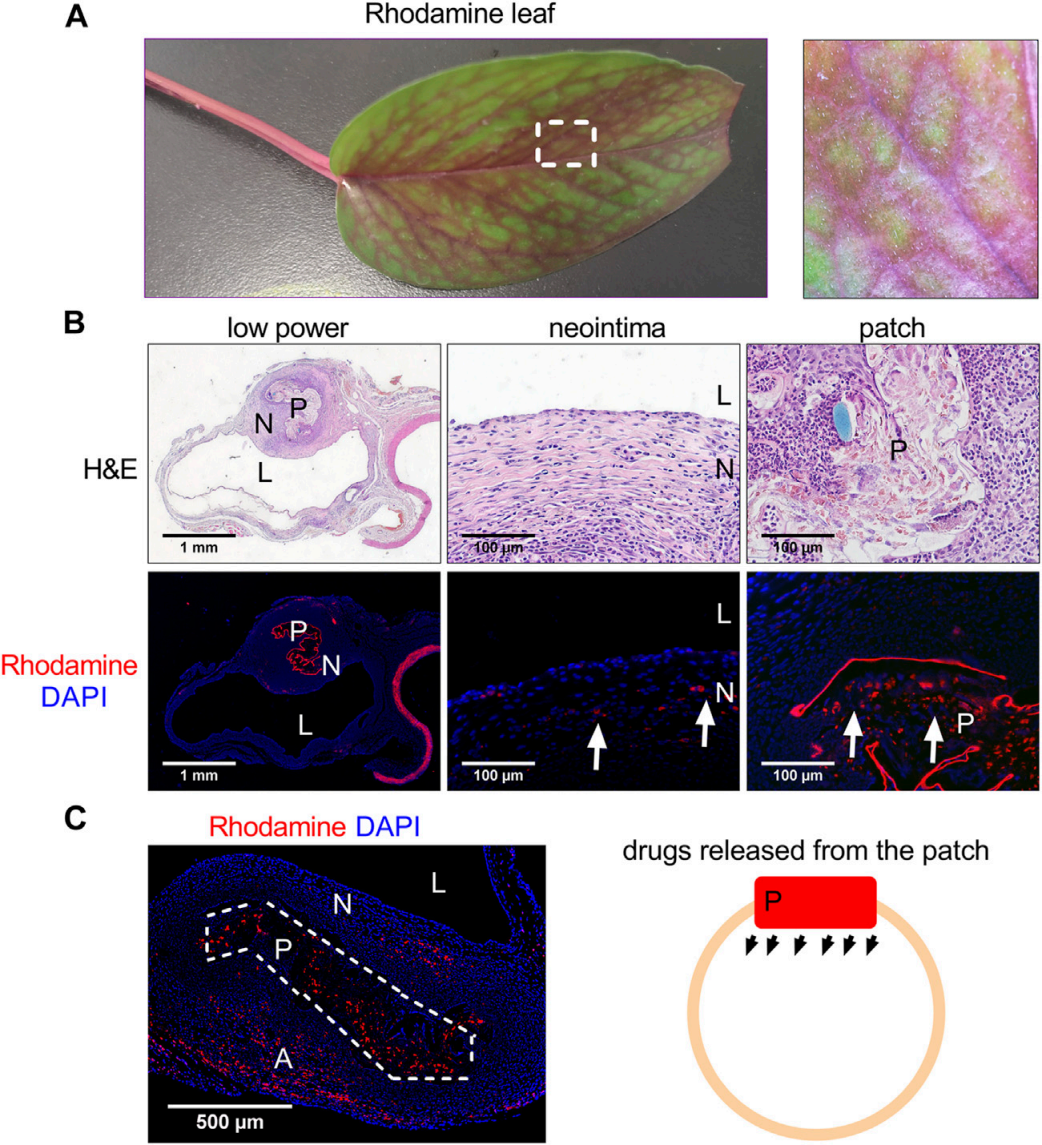
Photographs showing the plant leaf after absorption of rhodamine water. **(A)** Photograph showing the plant leaf after absorption of rhodamine water at day 2. **(B)** Low and high-power photographs of hematoxylin and eosin (H&E) staining of the rhodamine plant leaf patch harvested at day 14, L, lumen; N, neointima; P, patch; scale bar, 1 mm or 100 μm; *n* = 3. **(C)** Immunofluorescence photographs showing the rhodamine plant leaf patch harvested at day 14, L, lumen; N, neointima; P, patch; scale bar, 1 mm or 100 μm; white arrow showing the rhodamine fluorescence; *n* = 3. **(C)** Immunofluorescence photograph and illustration photograph showing the rhodamine released from the patch, L, lumen; N, neointima; P, patch; A, adventitia; scale bar, 500 μm; white dashed line area showing the patch; *n* = 3.

We first examined the absorption capability of fresh plant leaves. The leaf tip was cut to enhance the velocity of the absorption, then the leaf was put into rhodamine water (Figure 2). The leaf started to turn red from the leaf stem and then the small vascular at 6 h, after 24 h, the leaf had almost completely turned red (Figure 2A). After 48 h absorption, the rhodamine color was almost evenly distributed, the leaf (the evenly distributed part) was cut into patches (3 × 1.5 mm) and implanted into rat IVC; the patches were harvested at day 14 and analyzed (Figure 2). We then examined the release capability of rhodamine plant leaf *in vivo*. The patch was implanted into the rat IVC and harvested at day 14 (Figure 1). After 14 days, H&E staining showed that a neointima had formed on the luminal side, high power photographs showed that cells had migrated into the plant leaf patch (Figure 2B). Immunofluorescence showed the rhodamine fluorescence of the patch, there was rhodamine fluorescence in the neointima, inside the patch, and in the adventitia (Figures 2B,C), this result showed that the drugs could not only be absorbed by the plant leaf, but could also be released from the plant leaf gradually *in vivo* (Figure 2C).

**FIGURE 3:**
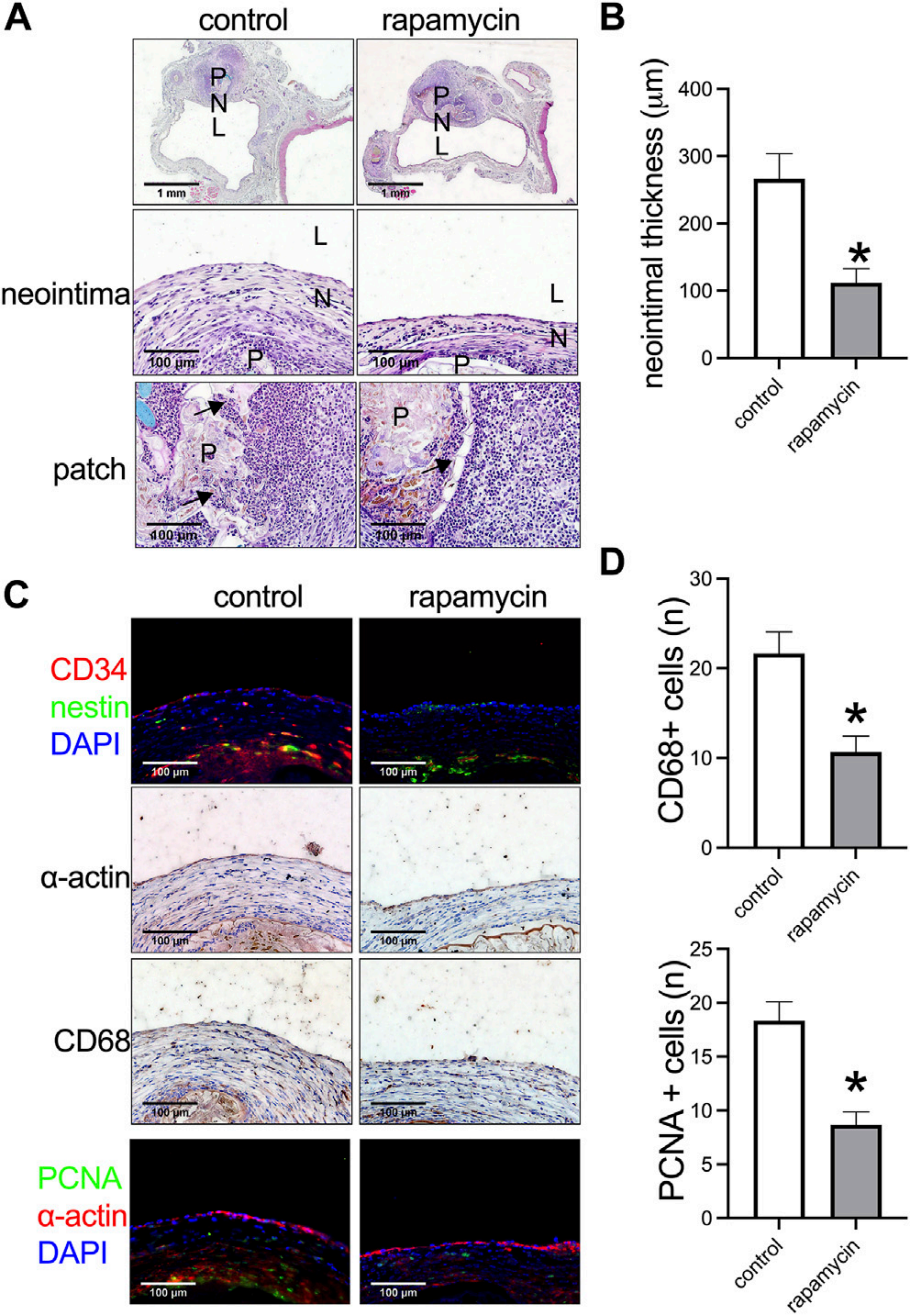
Rapamycin-absorbed plant leaf decreased neointimal thickness and neointimal hyperplasia after patch venoplasty in rats. **(A)** Photographs of the patch stained with hematoxylin and eosin (H&E) at day 14; P, patch; L, lumen; N, neointima; *n* = 3–5. **(B)** Bar graph showing the venoplasty neointimal thickness, *, *p* = 0.0231, t-test, *n* = 3. **(C)** Immunofluorescence stained for CD34 (red), nestin (green), and DAPI (blue) in the neointima; immunohistochemistry stained for α-actin and CD68 in the neointima; immunofluorescence stained for PCNA (green), α-actin (red), and DAPI (blue) in the neointima; scale bar 100 μm; *n* = 3. **(D)** Bar graphs showing the CD68 positive cells (*, *p* = 0.0210, t-test) and PCNA positive cells in the neointima (*, *p* = 0.0106, t-test); *n* = 3.

Rapamycin can inhibit venous neointimal hyperplasia (Bai et al., 2017a). We further examined whether a plant leaf patch absorbed with rapamycin could inhibit venous neointimal hyperplasia compared to the control patch. At day 14, after both patches were incorporated into the IVC, a neointima had formed in both groups, and cells had also migrated into the patches in both groups, but there was a significantly thinner neointima in the plant patch absorbed with rapamycin (Figures 3A,B). Progenitor cells participate in the process of neointimal formation after vascular surgery, CD34 positive cells are either hematopoietic or endothelial progenitor cells; nestin positive cells take part in developing and regenerating vasculature (Bai et al., 2020b; Bai et al., 2020c). There were CD34 and nestin positive cells in the neointima in both the control and rapamycin groups (Figure 3C), CD34 and nestin positive cells had also migrated to the patch (Supplementary Figure S1); there were α-actin (smooth muscle cell marker) and CD68 (macrophage marker) positive cells in the neointima (Figure 3C), with significantly fewer CD68 positive cells in the neointima of the rapamycin group compared to the control group (Figure 3D). We also examined inflammatory cells infiltration in the peri-patch area, CD3 (lymphocyte marker), CD68, and CD45 (leukocytes markers) positive cells were found in the peri-patch area in both the control and rapamycin groups (Supplementary Figure S1). Immunofluorescence showed there were significantly fewer PCNA (proliferation marker) positive cells in the neointima in the rapamycin group compared to the control group (Figures 3C,D). This result also showed that a plant leaf absorbed with rapamycin is effective and can be used as a novel drug delivery system.

**FIGURE 4:**
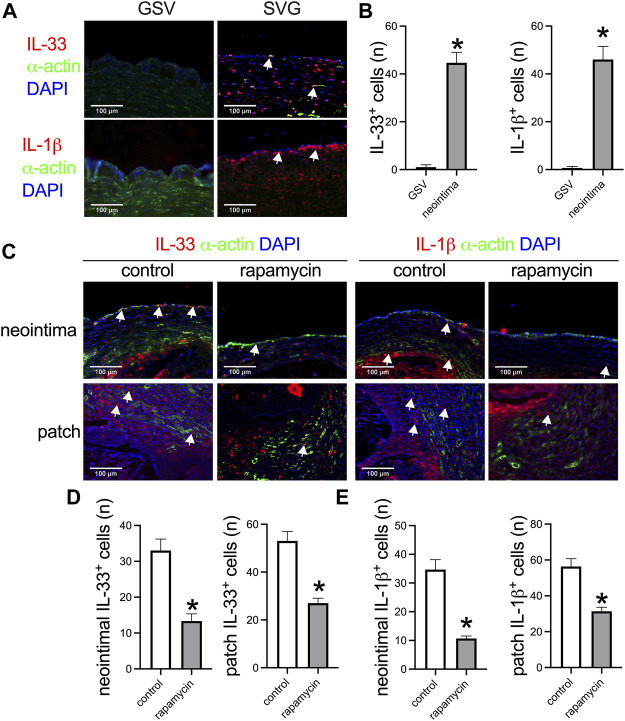
Higher IL-33 expression correlated with thicker venous neointimal hyperplasia both in humans and rats. **(A)** Immunofluorescence stained for IL-33 (red), α-actin (green), and DAPI (blue); IL-1β (red), α-actin (green), and DAPI (blue) in the human great saphenous vein (GSV) and spiral vein graft (SVG) neointima; white arrow showing the positive cells; *n* = 3. **(B)** Bar graphs showing the IL-33 positive cells (*, *p* = 0.006, t-test) and IL-1β positive cells in the neointima (*, *p* = 0.012, t-test); *n* = 3. **(C)** Immunofluorescence stained for IL-33 (red), α-actin (green), and DAPI (blue); IL-1β (red), α-actin (green), and DAPI (blue) in the control and rapamycin-absorbed leaf neointima and patch; white arrow showing the positive cells; *n* = 3. **(D)** Bar graphs showing the neointimal IL-33 positive cells (*, *p* = 0.0066, t-test) and patch IL-33 positive cells (*, *p* = 0.0046, t-test), *n* = 3. **(E)** Bar graphs showing the neointimal IL-1β positive cells (*, *p* = 0.0026, t-test) and patch IL-1β positive cells (*, *p* = 0.0073, t-test), *n* = 3.

IL-33 is a new member of the IL-1 family and plays a role in neointimal hyperplasia after artery injury (Govatati et al., 2019). We examined whether IL-33 was expressed in the venous neointima in the human sample. There were no IL-33 and IL-1β positive cells in the human great saphenous vein (GSV), but there was a significantly large number of IL-33 and IL-1β positive cells in the SVG neointima (Figures 4A,B). In the control and rapamycin-absorbed groups in rats, there were IL-33 and IL-1β positive cells in both groups in the neointima and in the peri-patch area (Figure 4C); but there were significantly fewer IL-33 and IL-1β positive cells in the rapamycin-absorbed group in both the neointima and peri-patch area (Figures 4C–E). These data showed that IL-33 plays a role in venous neointimal hyperplasia in both humans and rats.

**FIGURE 5:**
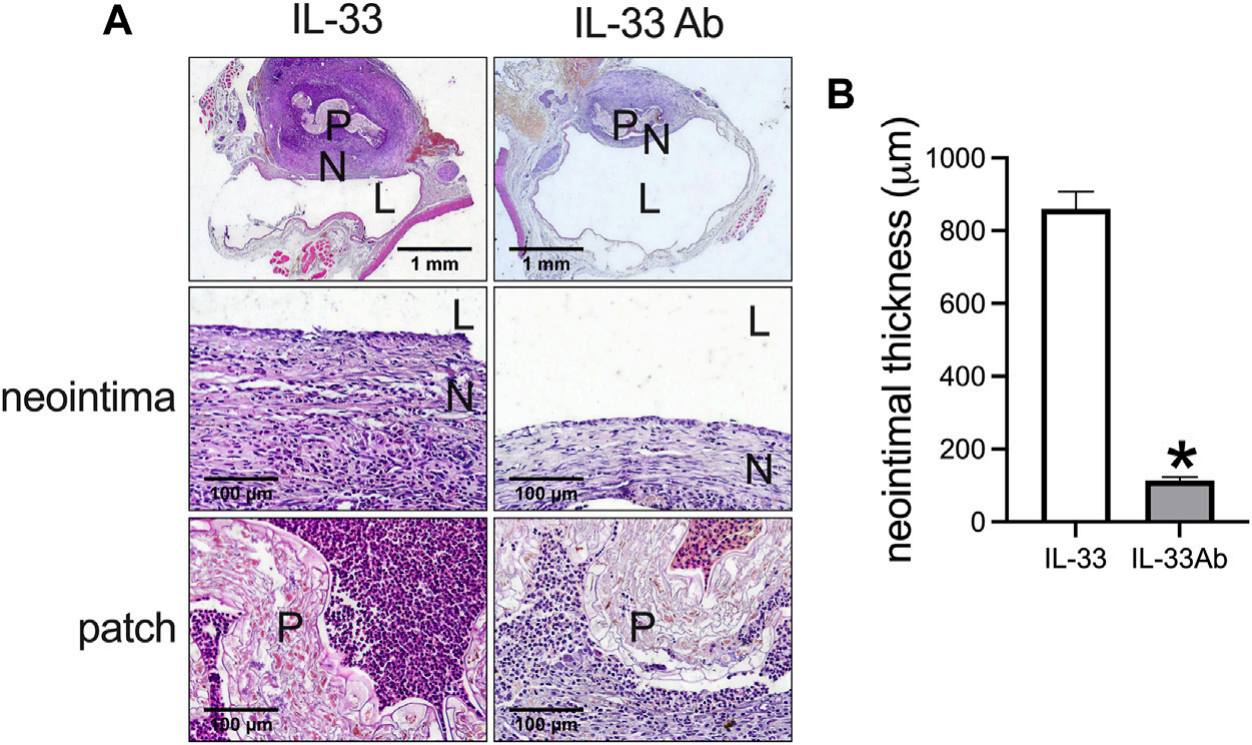
IL-33-absorbed plant leaf increased venous hyperplasia while IL-33 antibody (IL-33 Ab)-absorbed plant leaf decreased venous hyperplasia in the rat inferior vena cava (IVC) venoplasty model. **(A)** First row, photographs of hematoxylin and eosin (H&E) staining showing the IL-33 and IL-33 Ab-absorbed plant patch harvested at day 14; second row, high power showing the neointima; third row, high power showing the patch; P, patch; N, neointima; L, lumen; scale bar, 1 mm and 100 μm. **(B)** Bar graphs showing the neointimal thickness (*, *p* = 0.0001, t-test), *n* = 3.

**FIGURE 6:**
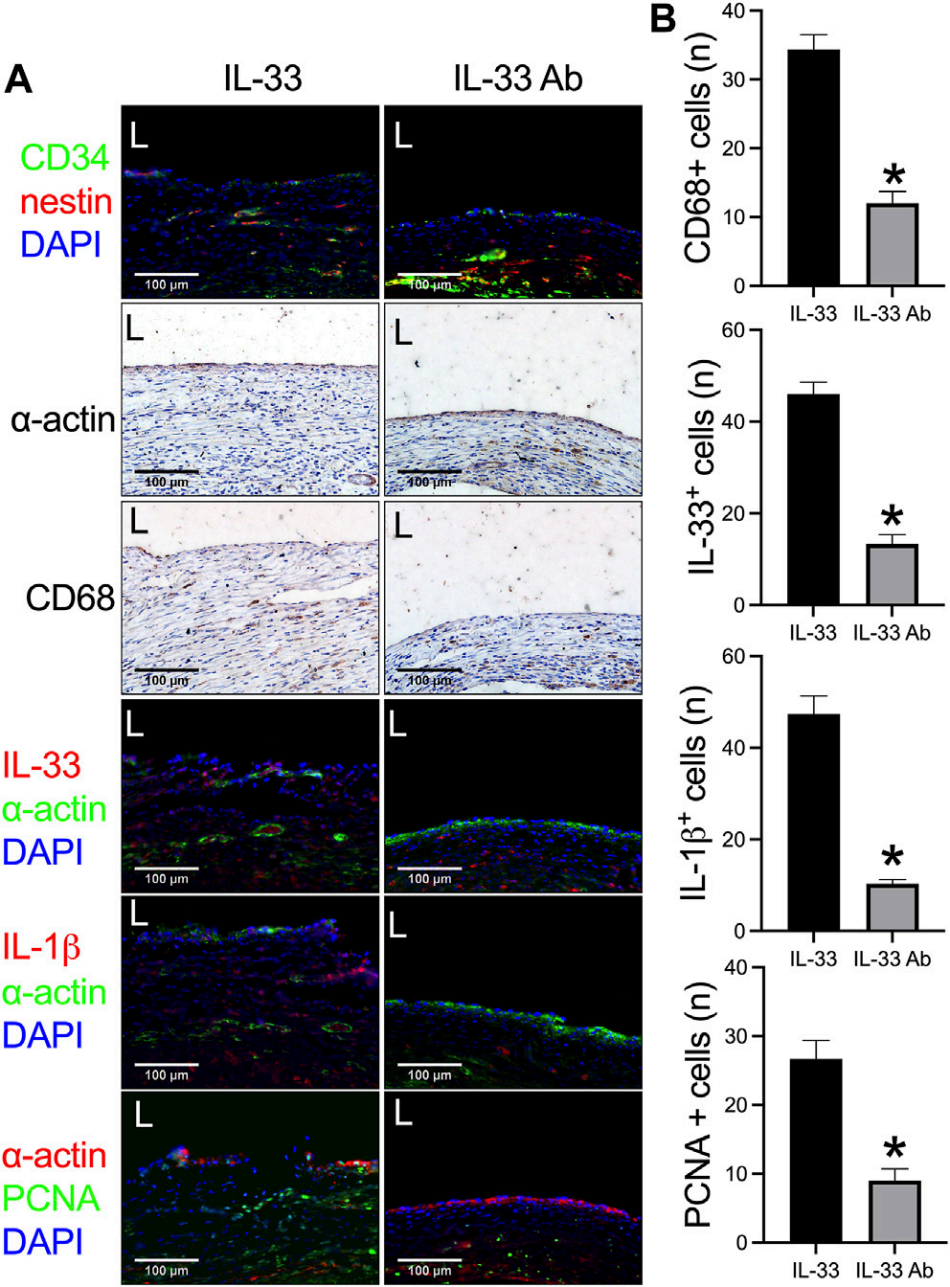
IL-33 antibody (IL-33 Ab)-absorbed plant leaf can decrease IL-33 expression and neointimal hyperplasia in a rat inferior vena cava (IVC) venoplasty model. **(A)** First row, immunofluorescence stained for CD34 (red), nestin (green), and DAPI (blue) in the neointima; second and third rows, immunohistochemistry stained for α-actin and CD68 in the neointima; fourth to sixth rows, immunofluorescence stained for IL-33 (red), α-actin (green), and DAPI (blue); IL-1β (red), α-actin (green), and DAPI (blue); and PCNA (green), α-actin (red), and DAPI (blue) in the IL-33 and IL-33 Ab-absorbed plant patch; L, lumen; scale bar 100 μm; *n* = 3. **(B)** Bar graphs showing the CD68 positive cells (*, *p* = 0.0013, t-test), IL-33 positive cells (*, *p* = 0.0006, t-test), IL-1β positive cells (*, *p* = 0.0008, t-test), and PCNA positive cells in the neointima (*, *p* = 0.0054, t-test); *n* = 3.

Then we examined whether neutralizing IL-33 with IL-33 antibody could decrease venous neointimal thickness in rats. Neutralizing antibodies is a widely used method to inhibit the function of the target antigen; program death-1 (PD-1) antibody can neutralize PD-1 function to decrease neointimal hyperplasia and aneurysm formation in rats (Bai et al., 2021b; Sun et al., 2021a; Sun et al., 2021b), systemic blockade of TGF-β by neutralizing antibodies can accelerate abdominal aortic aneurysm development in angiotensin II-infused mice (Angelov et al., 2017). We used IL-33 to increase and IL-33 neutralizing antibody to decrease the function of IL-33. The IL-33-absorbed plant leaf and IL-33 antibody-absorbed plant leaf patches were implanted into the rat IVC, the patches were harvested at day 14 and analyzed. There was a significantly thicker neointima in the IL-33-absorbed leaf patch group while a significantly thinner neointima was found in the IL-33 antibody-absorbed leaf patch group (Figures 5A,B). In both groups, there were CD34 and nestin positive cells in the neointima (Figure 6A). There were α-actin positive cells and CD68 positive cells in the neointima and in the peri-patch area, with significantly fewer CD68 positive cells in the IL-33 antibody-absorbed group compared to the IL-33-adsorbed group (Figure 6A, Supplementary Figure S2). There were also CD3 and CD45 positive cells in the neointima and in the peri-patch area in both groups (Supplementary Figure S2). We also examined the IL-33 and IL-1β expression in the IL-33 and IL-33 antibody-absorbed groups. There was a significantly larger number of IL-33 and IL-1β positive cells in the IL-33 group compared to the IL-33 antibody-absorbed group in the neointima (Figures 6A,B); there was also a significantly smaller amount of PCNA positive cells in the IL-33 antibody-absorbed group compared to the IL-33-absorbed group (Figures 6A,B). There were few cleaved caspase-3 positive cells in the neointima and peri-patch area in both groups (Supplementary Figure S2).

## Discussion

In this research, we showed that plant leaves can absorb drug solutions and show a homogenous distribution. A plant leaf absorbed with rapamycin can effectively inhibit venous neointimal hyperplasia in rats; IL-33 plays a role in venous neointimal hyperplasia both in humans and rats, and a plant leaf absorbed with IL-33 antibody can decrease venous neointimal hyperplasia in a rat venoplasty patch model.

From the autologous great saphenous vein graft to the commonly used prosthetic ePTFE and Dacron graft, each graft has its own merits and drawbacks (Bai et al., 2021c). Various modifications of these prosthetic grafts have been made, like heparin and rapamycin coating (Ambler and Twine, 2018; Baba et al., 2021) and collagen coating (Chou et al., 2017); these modification have contributed to the progress of vascular surgery. Along with the development of material science, new materials need to be tested for their application in vascular surgery. Various novel materials and grafts have been tested in vascular basic research or are being tested in clinical research (Niklason and Lawson, 2020; Bai et al., 2021d). Plants are green materials and are available almost anywhere, but the focus on plant-derived scaffolds has only been initiated in recent years, and only dozens of research papers have been published (Bai et al., 2021a). *In vitro* experiments show the promising potential application of plant-derived scaffolds. (Gershlak et al., 2017; Robbins et al., 2020) All of the research papers are *in vitro* experiments except our preliminary research (Lacombe et al., 2020; Jansen et al., 2020; Bai et al., 2021a). Compared to current prosthetic grafts, plant-derived scaffolds are widely available, much cheaper, and are free of animal-transmitted disease. But in our previous research, there were limitations in the drug delivery method. We injected the PLGA nanoparticle rapamycin hydrogel through the leaf stem, but the hydrogel could not equally distribute through the whole leaf, there was more of the drug near the leaf stem but less further away from the leaf stem; the pressure of the hydrogel injection may also destroy the native structure and vascular of the leaf. Based on the adsorption capacity of the leaf, it could directly absorb the drug solution and showed an equal distribution pattern after day 1. This leaf absorption method also has other merits, unlike the coating method where the coated drugs can detach from the balloon after the balloon is inflated. We previously performed nanoparticle rapamycin and TGF beta 1-coated pericardial patch angioplasty both in rat IVC and aorta, but there was little rhodamine left after 24 h (Bai et al., 2017a; Bai et al., 2018;). In this novel plant leaf absorption method, there was still lots of rhodamine left in the patch at day 14, which was released to the neointima and adventitia from the patch. This method is an efficient method to deliver drugs compared to our previous coating method (Bai et al., 2017a; Bai et al., 2018). The leaf was not decellularized in this research, since a decellularized leaf does not have an absorption capability like a fresh leaf.

Interleukin-33 (IL-33) is a new cytokine belonging to the IL-1 family, it plays an important role in cancers and human immunopathology (Nishida et al., 2010; Manetti et al., 2010). Recently, in a mouse guidewire injury model, Govatati et al. (2019) found that guidewire injury induces IL-33 expression and its neutralizing antibodies substantially reduces neointimal growth *in vivo*, they concluded that injury-induced neointimal growth requires IL-33 expression. In a previous human spiral saphenous vein graft harvested from the popliteal vein, there was IL-33 and IL-1β expression in the neointima, but there were no IL-33 and IL-1β positive cells in the human saphenous vein (Figure 4). In the neointima of the rat IVC venoplasty, there was also IL-33 and IL-1β in the neointima, with significantly fewer IL-33 and IL-1β positive cells in the rapamycin group; this result showed that neutralization of IL-33 may be a therapeutic method to decrease venous neointimal hyperplasia. We also confirmed this hypothesis using IL-33 and IL-33 antibody, there was a significantly thicker neointima in the IL-33 patch group compared to the IL-33 antibody patch group. This result showed that plant leaves cannot only can be used as a venous patch but can also be an efficient drug delivery system. Human autologous great saphenous vein grafts are commonly used in vascular reconstruction (Bai et al., 2020a), we previously showed that the human neointimal formation process shares similarities with the same process in rats (Sun et al., 2021c). Patch angioplasty is a useful model to explore the mechanism and treatment method of neointimal hyperplasia, (Bai et al., 2020b; Bai et al., 2021b) and different patches shared a similar healing process (Bai et al., 2021c). We also showed that plant-derived patches can be used as venous patches in the rat IVC venoplasty model (Bai et al., 2021a), this previous research led us to use the novel leaf patch to examine the role of IL-33 in venous neointimal hyperplasia. IL-33 has a commercially available antibody known as etokimab, so this research has a potential clinical application. (Chinthrajah et al., 2019; Chen et al., 2019).

We showed that cells can migrate to the plant leaf after implantation; other groups also showed a similar result *in vitro*. A decellularized apple scaffold is comparable to other natural and synthetic scaffold materials, cells can adhere, invade, and proliferate in the three-dimensional cellulose structure (Modulevsky et al., 2014). Plant-based scaffolds have many physical property advantages (Lacombe et al., 2020). Various decellularized fruit and vegetable -derived tissues as scaffolds have been investigated *in vitro* (Salehi et al., 2020; Contessi Negrini et al., 2020). Previously, there was a very complex foreign body reaction after implantation of foreign materials (Klopfleisch and Jung, 2017; Jurak et al., 2021), we also showed that foreign body reaction occurred after implantation of different patch materials. The new tissue capsuled the patch as early as day 7 after implantation (Bai et al., 2021c), the inflammatory response of surgical implantation deceased after 2 weeks, (de la Oliva et al., 2018), (Bai et al., 2017c) and the leaf patch also showed a similar healing process when compared to other patch materials (Bai et al., 2021a). In this research, the patch was capsuled by a new tissue induced by the foreign body reaction, macrophages infiltrated in the peri-patch area and mediated the foreign body reaction process (Figures 2, 3), this reaction was like the decellularized leaf and onion cellulose patch we showed previously (Bai et al., 2021a). The new tissue capsuled the plant patch to decrease further foreign body reaction, this made the plant patch tolerable in the animal body. Although the leaf patch was capsuled by fibrotic tissue, this foreign body reaction did not decrease the performance of the patch. The rhodamine released from the patch showed a wide range of distribution in the neointima, in the adventitia, and in the tissue surrounding the patch (Figure 2), it is possible that the use of a decellularized leaf can induce a lower foreign body reaction, so the leaf patch can still interact with the surrounding physiological environment because of this lower reaction. The nature leaf structure, the physical and chemical characteristics of the leaf, and the nature leaf surface may contribute to this decreased foreign body reaction. But the foreign body reaction of the plant material in an animal body needs further exploration. Many general mechanisms of the foreign body reaction have been elucidated, but the specific response to a biomaterial largely depends on its physical and chemical characteristics, especially of its surface (Klopfleisch and Jung, 2017). The surface properties of the biomaterials can directly influence the foreign body response; many other factors like implant design, implant localization, state of the host bed, surgical technique, and mechanical loading also influence this process (Klopfleisch and Jung, 2017). In one study, Modulevsky et al. (2016) created implantable cellulose scaffolds from apples that were subcutaneously implanted in wild-type, immunocompetent mice, and demonstrated that native cellulose scaffolds are biocompatible and exhibit promising potential as a surgical biomaterial. But further research on the foreign body reaction to plant biomaterial is still needed.

In conclusion, we found that the natural absorption capability of plant leaves means they can absorb drug solutions efficiently and can be used as a patch and a novel drug delivery system. IL-33 plays a role in venous neointimal hyperplasia both in humans and rats, and neutralization of IL-33 by IL-33 antibody can be a therapeutic method to decrease venous neointimal hyperplasia.

## Data Availability

The raw data supporting the conclusion of this article will be made available by the authors, without undue reservation.

## Supplementary Material

Supplementary Figure S1 Analysis of the cell composition change in the peri-patch area in the control and rapamycin-absorbed plant patch harvested at day 14, first row, immunofluorescence stained for CD34 (red), nestin (green), and DAPI (blue); second to fourth rows, immunohistochemistry stained for CD3, CD68, and CD45; scale bar 100 μm; *n*=3.

Supplementary Figure S2 Analysis of the cell composition change in the neointima and peri-patch area in the IL-33 and IL-33 antibody (IL-33Ab)-absorbed plant patch harvested at day 14, sections were stained for CD68, CD3, CD45, and cleaved caspase-3; scale bar 100 μm; *n*=3.
