# Supplementary figures and images for: A Novel Plant Leaf Patch Absorbed With IL-33 Antibody Decreases Venous Neointimal hyperplasia
Source: Front Bioeng Biotechnol. 2021 Oct 29;9:742285. doi: 10.3389/fbioe.2021.742285 (PMC8585764; doi:10.3389/fbioe.2021.742285)

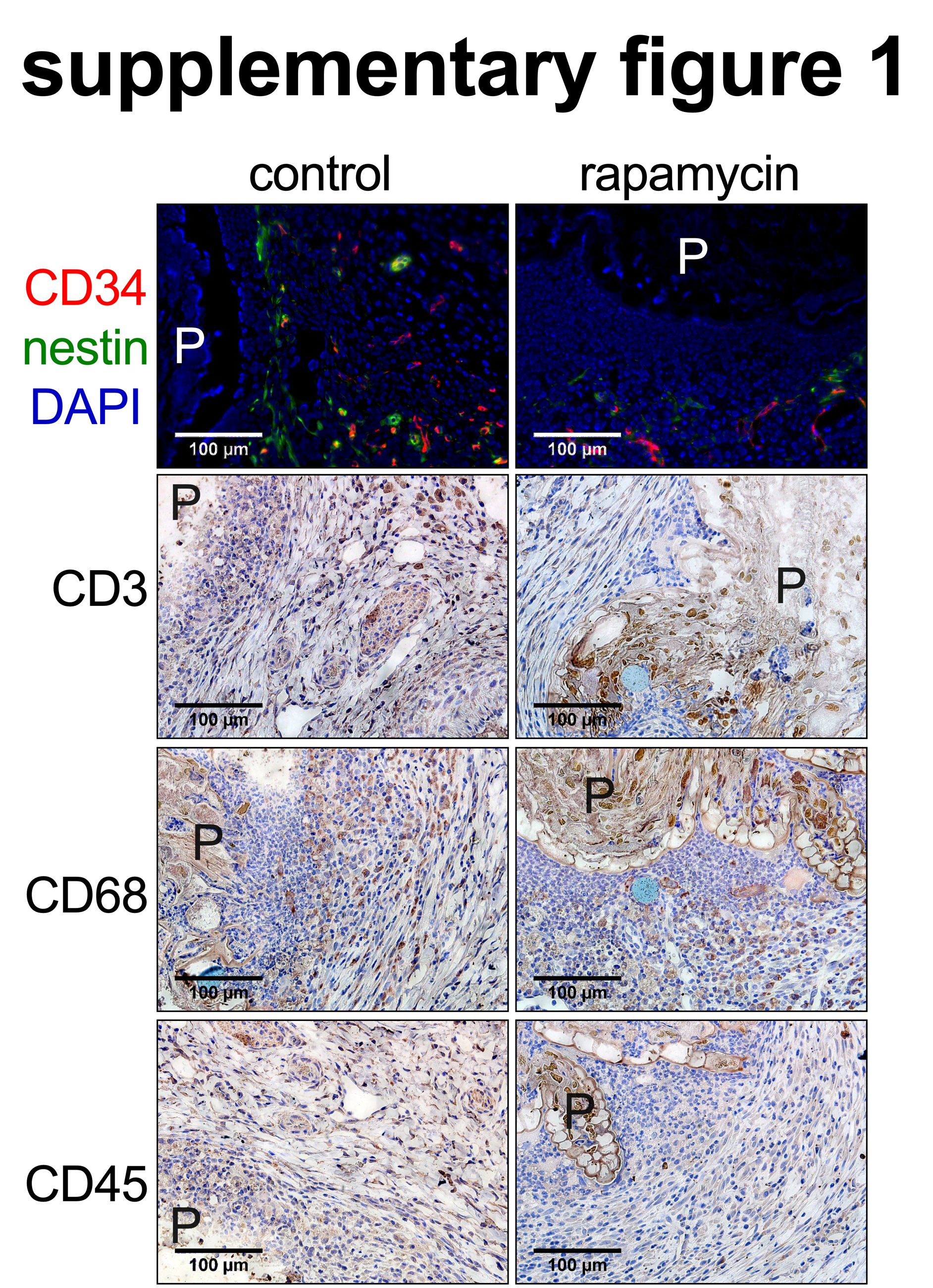

Supplement: Supplementary file 1 [file Image1.TIFF]

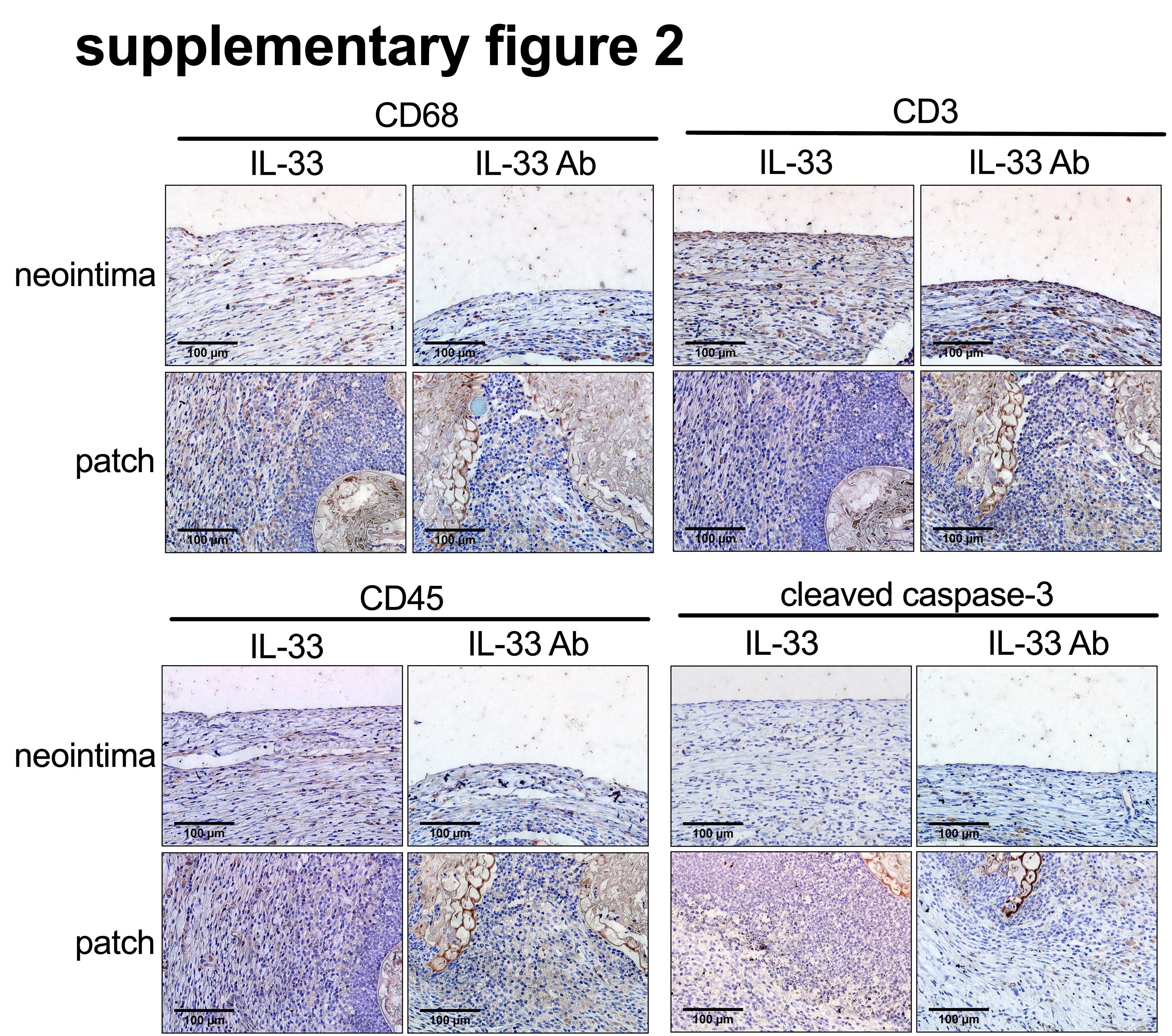

Supplement: Supplementary file 2 [file Image2.TIFF]
